# Adding insult to injury: the spectrum of tubulointerstitial responses in acute kidney injury

**DOI:** 10.1172/JCI188358

**Published:** 2025-03-17

**Authors:** Megan L. Baker, Lloyd G. Cantley

**Affiliations:** 1 Yale University School of Medicine, Department of Internal Medicine, Section of Nephrology, New Haven, Connecticut, USA

## Abstract

Acute kidney injury (AKI) encompasses pathophysiology ranging from glomerular hypofiltration to tubular cell injury and outflow obstruction. This Review will focus on the tubulointerstitial processes that underlie most cases of AKI. Tubular epithelial cell (TEC) injury can occur via distinct insults, including ischemia, nephrotoxins, sepsis, and primary immune-mediated processes. Following these initial insults, tubular cells can activate survival and repair responses or they can develop mitochondrial dysfunction and metabolic reprogramming, cell-cycle arrest, and programmed cell death. Developing evidence suggests that the fate of individual tubular cells to survive and proliferate or undergo cell death or senescence is frequently determined by a biphasic immune response with initial proinflammatory macrophage, neutrophil, and lymphocyte infiltration exacerbating injury and activating programmed cell death, while alternatively activated macrophages and specific lymphocyte subsets subsequently modulate inflammation and promote repair. Functional recovery requires that this reparative phase supports proteolytic degradation of tubular casts, proliferation of surviving TECs, and restoration of TEC differentiation. Incomplete resolution or persistence of inflammation can lead to failed tubular repair, fibrosis, and chronic kidney disease. Despite extensive research in animal models, translating preclinical findings to therapies remains challenging, emphasizing the need for integrated multiomic approaches to advance AKI understanding and treatment.

## Introduction

Acute kidney injury (AKI) is a syndromic term that is defined and stratified by changes in serum creatinine levels, reflecting reciprocal changes in glomerular filtration rate (GFR), with or without changes in urine output. This method of defining AKI is clinically useful, but encompasses countless distinct pathologic events in the glomerulus, tubules, interstitium, or ureter that result in reduced plasma filtration by the kidney and thereby complicates translation to patient-specific therapeutic intervention in clinical practice. AKI due to physiologically mediated changes in glomerular hemodynamics, such as with hypovolemia or renin-angiotensin-aldosterone system inhibitor or sodium-glucose cotransporter 2 inhibitor use, can rapidly recover when the cause is identified and addressed. Many of the glomerular pathologies associated with AKI, including immune-mediated glomerulonephritides, thrombotic microangiopathies, and vasculitides, have specific therapies that can slow or reverse the loss of GFR. However, the loss of GFR that accompanies intrinsic tubular injury has no specific therapy and frequently leads to prolonged AKI, the need for renal replacement therapy, and progression to chronic kidney disease (CKD), end-stage kidney disease, or death. In a study comparing animal models of decreased kidney function due to extracellular fluid volume contraction versus intrinsic kidney injury, RNA-Seq demonstrated that the thousands of induced genes between these two models comprised functionally unrelated signal transduction pathways expressed in different regions of the kidney, suggesting that these two forms of AKI induce distinct biological responses (1). The focus of this Review will be on forms of AKI in which kidney tubular epithelial cell (TEC) injury is the prominent defining feature and for which treatment strategies are lacking.

**Figure 1:**
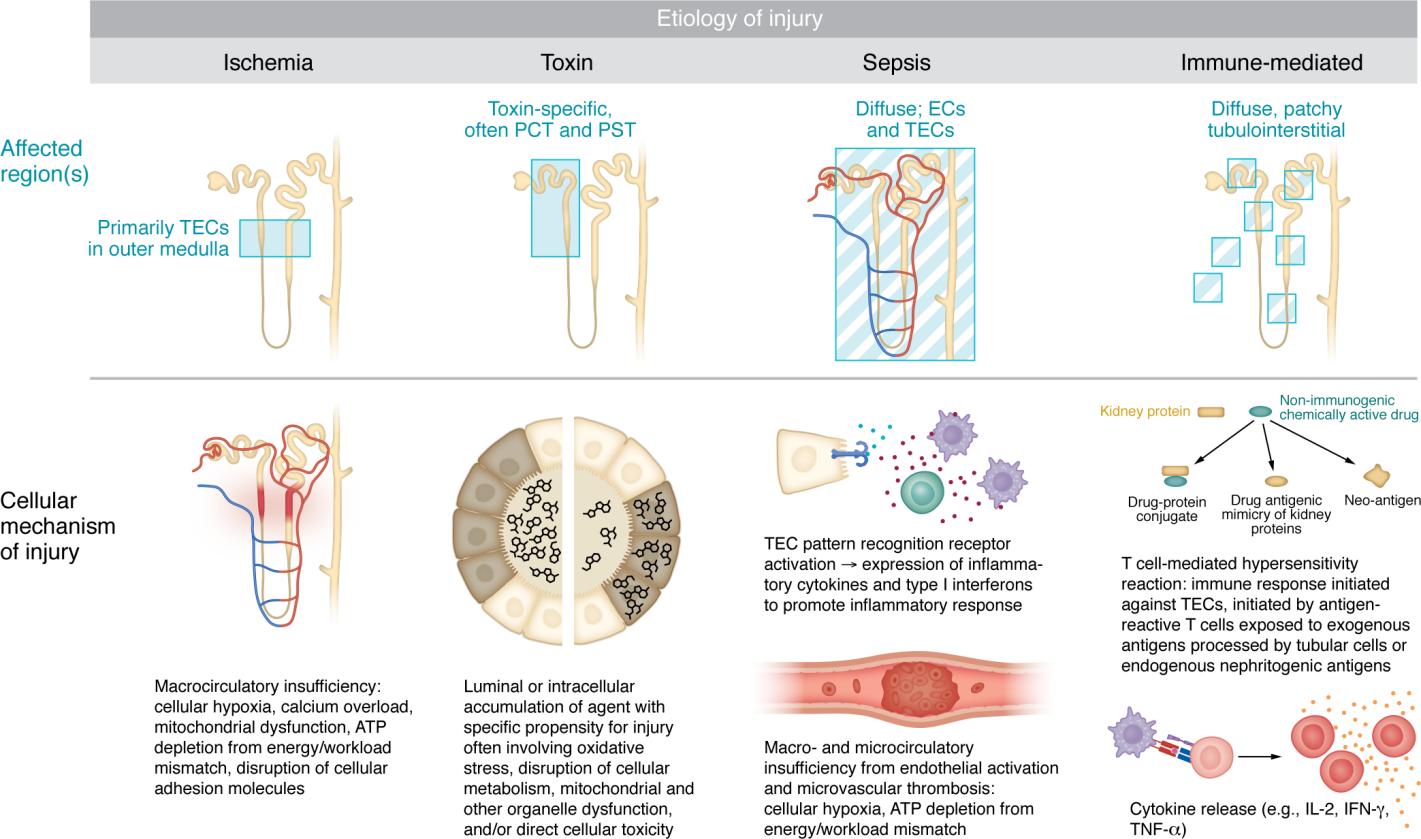
Patterns of epithelial cell injury in response to distinct injury stimuli. Defined classes of tubular insults can induce distinct initial mechanisms and distributions of cellular injury. From left to right, macrocirculatory insufficiency in ischemic injury results in mitochondrial dysfunction and cellular metabolic and energy disturbances. In toxin-mediated AKI, the cellular mechanisms of injury are dependent on toxin characteristics and toxin handling within the tubule (i.e., secretion or filtration and accumulation within tubular space or TEC absorption and intracellular accumulation). Septic AKI is characterized by endothelial injury and activation along with TEC injury resulting from both pattern recognition receptor activation on TECs as well as cellular energy and metabolic derangements from macro-and microcirculatory insufficiency. In immune-mediated injury such as AIN, antigens elicit a cell-mediated T cell hypersensitivity immune response either directly or after hydrolysis and processing by tubular cells to form a hapten bridge. PCT, proximal convoluted tubule; PST, proximal straight tubule.

**Figure 2:**
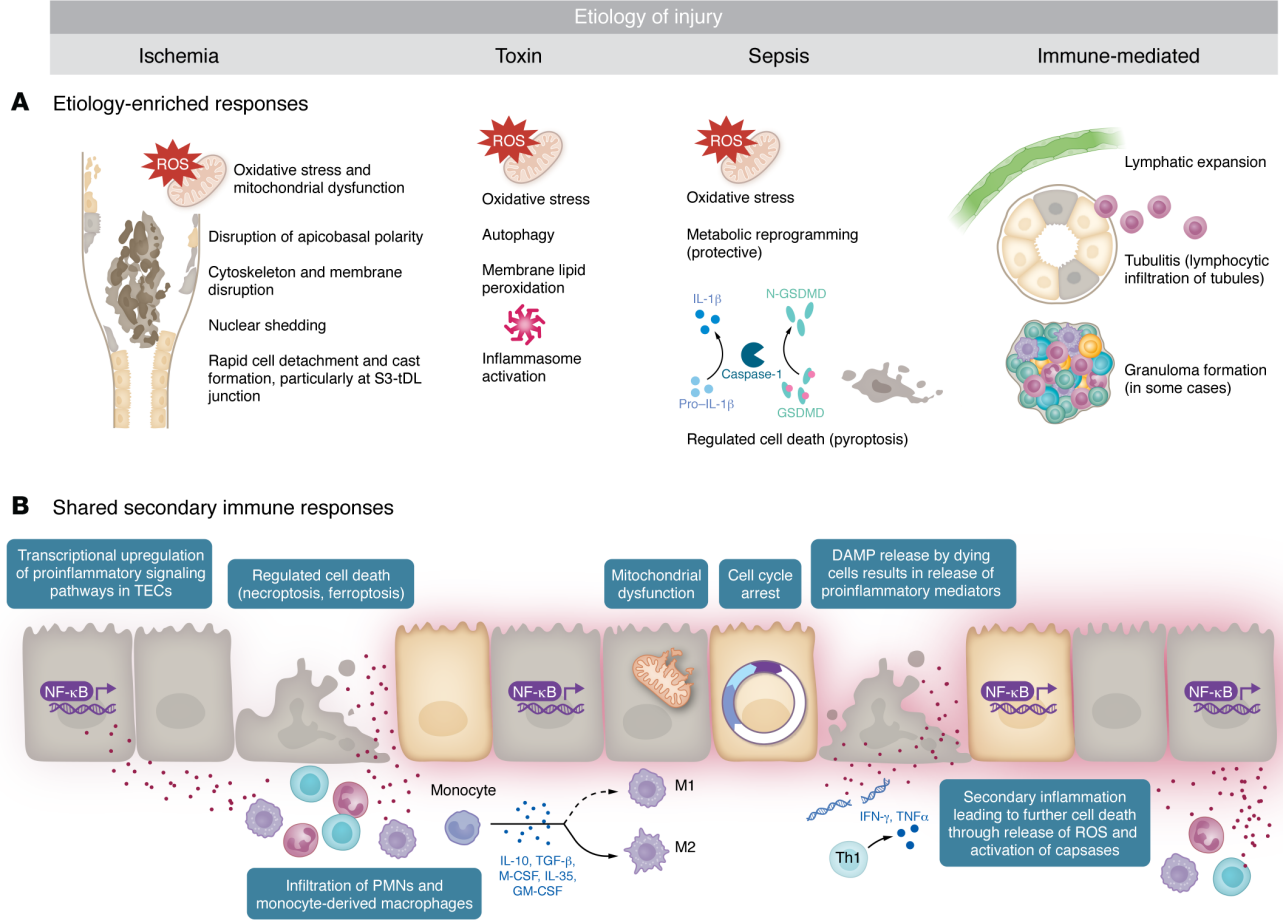
Surviving epithelial cell responses to distinct injury stimuli. (**A**) The repertoire of injury responses by tubular cells that survive the initial insult is limited and is at least in part determined by both the type and severity of injury. Primary responses such as metabolic reprogramming, inflammasome activation, and cast formation often predominate in specific types of initial injury. (**B**) However, many of the secondary immune responses are shared and can lead to cell-cycle arrest, PCD pathways, and recruitment of secondary immune cells.

TEC injury can result from a variety of pathophysiologic processes, and animal models have been developed to recapitulate and mechanistically study the cellular responses that underlie some of the associated clinical scenarios. While serum creatinine elevation defines all instances of AKI, experimental data suggest that the molecular and cellular responses following injury caused by differing stimuli are heterogenous and reflect distinct pathophysiological pathways (2). This is partly reflected by the measurement of renal injury biomarkers that exhibit differential expression patterns in response to distinct insults and the resulting distribution of injury throughout the nephron. Because their accuracy for predicting AKI differs depending on the clinical scenario and none are entirely specific to AKI, the currently validated biomarkers have been slow to gain acceptance in medical practice (3). While clinical use of specific biomarkers can aid in correct diagnosis and early detection of AKI, an equally urgent challenge is to break apart the “syndrome” of AKI into distinct, mechanistically aligned subgroupings that share the same underlying cellular drivers and responses (Figures 1 and 2) and then target those pathways for development of much-needed therapeutic discoveries.

## Inciting mechanisms of cellular injury

### Ischemic injury to tubular epithelium.

The most common form of tubular injury in patients occurs due to renal hypoperfusion resulting in TEC ischemia. The kidneys are susceptible to ischemia in large part due to the anatomy of their microcirculation. The cortex receives almost 100% of the blood flowing through the kidneys, whereas the medulla receives only 5%–10% in order to facilitate the process of urinary concentration (4). Proximal TECs (PTECs), the most abundant cell type in the cortex, extend into the outer stripe of the medulla and reabsorb the majority of electrolytes, minerals, glucose, proteins, and other macromolecules from the glomerular filtrate to maintain volume and solute homeostasis, a highly energy-dependent function (4–7). These cells rely on fatty acid oxidation for ATP production to meet their high metabolic demands and are thus highly susceptible to injury or death following reduction in blood flow (8, 9). In the 24–48 hours after severe kidney ischemia, extensive loss of TECs occurs due to both necrotic and programmed cell death (PCD) pathway activation. It remains unknown whether this also occurs in hemodynamic AKI in which no intrinsic injury is clinically documented, although adjudicated cases of hemodynamic AKI and acute tubular injury (ATI) display indistinguishable levels of multiple tubule injury biomarkers including KIM-1, NGAL, and IL-18 (10). Impaired blood flow can also cause injury to vascular endothelium and promote thrombosis, with severely injured peritubular vessels undergoing cell death that can cause peritubular rarefaction, an important contributor in the progression from AKI to the development of CKD (11–13).

Experimental animal models of renal ischemia-reperfusion injury (IRI) have been widely used to study the pathogenesis of ischemic AKI. These models initiate IRI through renal pedicle clamping and release, more closely approximating ischemia during kidney surgery or transplantation than renal ischemia resulting from hypotension or other causes of hypoperfusion. Transcriptional studies that have sought to characterize the validity of murine IRI as a model for human AKI showed clear differences in single-cell gene expression changes occurring during human ATI and mouse IRI, but also revealed substantial overlap for pathway-level changes that support the use of mouse IRI to identify mechanistic responses to ischemia and their cellular origin (14, 15).

It is widely accepted that the proximal straight tubule (S3) sustains the highest degree of injury after IRI (16–20), although the S1 segment is also susceptible to injury because of a lower capacity to generate ATP from glycolysis (21–23) (Figure 1). This general reliance on mitochondrial respiratory chain for sufficient ATP generation makes PTECs highly dependent on oxygen and nutrient delivery (4–7). In the absence of sufficient blood flow, PTECs can quickly develop severe ATP depletion leading to membrane disruption, nuclear shedding, calcium influx, and cell detachment (2, 19, 24). PCD pathways including apoptosis and regulated necrosis are believed to be the primary forms of cell death in AKI (24, 25). Inhibition of apoptosis protects against tubular cell death and reduces kidney function decline following AKI in animal models (25). ATP depletion, oxidative stress, secondary inflammation, and cellular hypoxia are all drivers of PCD in this setting (2, 26).

After injury, detached TECs can be cleared as nonocclusive urinary debris or can aggregate into casts that obstruct the tubule lumen and further reduce GFR (16, 27) (Figure 2). Cellular debris resulting from membrane rupture and cell death in S3, along with tubule narrowing at the S3-thin descending limb (S3-tDL) junction, makes S3 a common site of formation of occlusive casts (19, 28). As discussed below, TLRs expressed on surviving cells detect cellular debris, inducing a secondary immune response that appears to critically determine long-term outcomes for the injured tubule. Interactions between mislocalized proteins on the surface of detached cells within casts and proteins on surviving cells may also serve to anchor casts within the tubular lumen (29). Markedly increased cell detachment at S3 compared to tDL creates an expansion of the distal S3 lumen, exaggerating the bottleneck at the S3-tDL junction (19). In a murine IRI model, tracking of cast formation and movement through multiphoton imaging revealed that visually occlusive casts first appear 12 hours after IRI at the S3-tDL junction and peak at 24 hours with occlusive casts in 99% of S3 and 78% of tDL segments (19). By day 3 after IRI, while more distal nephron segments were cast free, 72% of S3 tubules and 58% of tDL tubules still contained occlusive casts (19). Clearance of these casts by phagocytosis and proteolysis, along with regeneration of the lost TECs, appears to be the tipping point that determines whether that tubule undergoes functional repair or progressive atrophy and serves as a nidus for chronic inflammation (19, 30).

### Toxic injury to tubular epithelium.

Nephrotoxin injury to the tubulointerstitium is another common form of AKI. The propensity for toxic injury of TECs is linked to their unique ability to reabsorb large amounts of some components of the glomerular filtrate while concentrating other components in the urinary space. This can lead to either toxic luminal concentrations of substances that are not reabsorbed (e.g., oxalate) or toxic intracellular concentrations of substances that are absorbed (e.g., lead, gentamycin) (31, 32) (Figure 1). Toxic injury is typically not limited to the proximal tubule and occurs through a combination of oxidative stress, autophagy, cell-cycle arrest, membrane-lipid peroxidation, and lumen obstruction, ultimately leading to PCD rather than cell necrosis (33). The list of exogenous compounds demonstrated to be toxic to TECs encompasses numerous therapeutic agents, intoxicants, contrast media, and environmental exposures. In recognition of this, the FDA approved a safety biomarker panel in 2018 comprising six biomarkers (cystatin-C, KIM-1, NGAL, NAG, osteopontin, clusterin) to improve detection of renal TEC injury caused by medications undergoing phase I clinical trials (34). Endogenous biomolecules represent a second category of nephrotoxins. Overproduction or excessive release of many molecules that are otherwise nontoxic can result in ATI, including uric acid in tumor lysis syndrome, myoglobin in rhabdomyolysis, and paraproteins in myeloma and other bone marrow dyscrasias. Like the responses seen with exogenous toxins, many endogenously generated toxins induce ATI via membrane injury, oxidative stress, and secondary immune activation, leading to PCD via regulated pathways such as necroptosis and ferroptosis (35, 36).

### Septic injury to tubular epithelium.

Sepsis is characterized by dysregulated activation of the immune system caused by the release of pathogen-associated molecular patterns (PAMPs) such as lipopolysaccharide by the infecting organism, and damage-associated molecular patterns (DAMPs) including proteins, lipids, and DNA from injured cells (37). Systemically, this can lead to depressed cardiac contractility, vasodilation, and hypotension, with renal hypoperfusion and ATI as discussed above (38). The dysregulated inflammatory response can heighten the secondary immune response to this hypotensive cellular injury, but can also induce tubular injury even in the absence of hypotension. An individual patient’s resulting phenotype of septic AKI depends heavily on their underlying susceptibility, leading to a variety of syndromic endotypes that the clinical presentation cannot easily distinguish (37). This complicates the identification of sepsis-induced AKI in the absence of clinical tools, such as biomarkers, to distinguish the etiology (37). Since biopsy is not frequently performed during sepsis, most of our current understanding of sepsis-induced AKI has been extrapolated from animal models, in vitro cellular studies, and postmortem observations in septic humans (39, 40).

Three mechanisms are consistently identified across injured organ systems during sepsis: inflammation, microcirculatory dysfunction, and metabolic reprogramming (41). The inflammatory response is essential for defending the body against pathogens, but when dysregulated can lead to organ dysfunction (42). PAMPs and DAMPs bind to TLRs on immune cells and TECs, triggering a cascade of signals that produce proinflammatory molecules and renal tubular dysfunction (43, 44). In renal TECs, particularly those expressing TLR2 and TLR4, this results in increased oxidative stress and mitochondrial injury (45, 46). TLR expression was markedly upregulated in all nephron segments in response to sepsis in an animal model (47).

Experimental and clinical studies have demonstrated that even in the absence of macrohemodynamic instability, microcirculatory alterations develop during sepsis through both reduced capillary density and disrupted blood flow and likely play a key role in the development of organ injury (48, 49). Endothelial cell (EC) injury, autonomic nervous system dysregulation, shedding of the glycocalyx, and activation of the coagulation cascade all contribute to microcirculatory alterations in sepsis (50, 51). EC injury and glycocalyx shedding facilitate leukocyte and platelet adhesion, reducing blood flow velocity and increasing the risk of microthrombi formation. This can cause capillary occlusion and prolonged exposure of TECs to inflammatory mediators, leading to vasodilation, increased vascular permeability, and interstitial edema, which impairs TEC perfusion by increasing oxygen diffusion distance and altering convection (48, 51) (Figure 1). Both sluggish flow and increased expression of intercellular adhesion molecule 1 (ICAM-1) and vascular cell adhesion molecule 1 (VCAM-1) in peritubular capillaries prolong leukocyte transit and increase paracrine signaling with kidney dendritic cells (52–54). Overall, prolonged cellular transit time may translate into longer exposure of endothelium and TECs to activated, cytokine-secreting leukocytes, PAMPs, and DAMPs, leading to amplification of the inflammatory signal and greater oxidative stress (41).

During sepsis, metabolic reprogramming in TECs shifts energy use to prioritize cell survival. This involves a mitochondrial-mediated process that optimizes energy expenditure, alters substrate utilization, and counters proapoptotic triggers (55, 56). TECs shift from oxidative phosphorylation to aerobic glycolysis to adapt to the septic environment (55, 57). Maintaining functional mitochondria through processes like mitophagy and biogenesis is critical for cell survival, as these organelles are central to energy production and metabolic reprogramming. In a study comparing high-quality microarray studies of renal gene expression of AKI in 6 different AKI disease models, AKI induced by gram-negative sepsis had the largest number of uniquely regulated genes as compared to other mechanisms of AKI, specifically in mitochondrial genes (2).

Cell-cycle arrest is another protective mechanism TECs employ to conserve energy during sepsis. By halting replication, cells avoid death due to ATP depletion. Markers of cell-cycle arrest, such as TIMP-2 and IGFBP7, have been identified as potential predictors of sepsis-induced AKI, underscoring the importance of this mechanism in human sepsis (58). TECs may also initiate paracrine signaling to neighboring cells to limit cell death, albeit at the expense of reabsorptive function (41).

### Primary immune-mediated injury to tubular epithelium.

Acute interstitial nephritis (AIN) is a form of AKI characterized by an idiosyncratic delayed hypersensitivity immune reaction that directly, and often selectively, injures TECs. In contrast to ischemic, sepsis-induced, and toxic AKI where tubular injury drives secondary inflammation, inflammation is the primary driver of injury in AIN. The immune response is initiated by antigen-reactive T cells exposed to exogenous antigens processed by TECs or endogenous nephritogenic antigens (59) (Figure 1). In over 75% of cases of biopsy-proven AIN, a drug serves as the inciting antigen, with infection-associated antigens (5%–10%) and autoimmune responses to endogenous proteins (10%–15%) accounting for most of the remainder.

Multiple mechanisms have been identified by which inciting antigens elicit a cell-mediated immune response, including molecular mimicry, serving as a hapten bridge to modify the immunogenicity of native kidney proteins, and toxic injury to the tubulointerstitium producing nephritogenic neoantigens (60–62). Resident peritubular mononuclear phagocytic cells (dendritic cells and macrophages) or injured TECs then function as antigen presenting cells (APCs), expressing antigenic components as peptides located on their surface MHC II molecules (63, 64). Activated APCs can migrate through the kidney lymphatic vessels to regional draining lymph nodes where they present the target antigen to naive T cells, which clonally expand to generate an activated T cell repertoire, including effector T cells that enter the circulation to home back to the kidney. The critical importance of these activated T cells in the pathogenesis of AIN is underscored by the clinical prevalence of AIN in patients taking immune checkpoint inhibitors (ICIs) to activate T cell responses to tumor antigens in the treatment of some cancers (65). ICIs can either promote the development of AIN in response to previously tolerated drugs (e.g., NSAIDs and H2 blockers) or induce de novo AIN in the absence of other known drug precipitants, potentially as an autoimmune response to endogenous antigens (66). Tubulitis, characteristic of severe AIN, is a focal lesion where inflammatory cellular infiltrates penetrate the tubular basement membrane with injury to the basolateral surface of adjacent TECs, and likely relies on the presence of target antigen on the TEC itself.

Effector T cells produce injury through two main mechanisms: the release of inflammatory cytokines to facilitate downstream immune responses and direct cell-mediated cytotoxicity via secreted proteases (67–69). One subset of effector T cells, designated Th9, produce IL-9, which leads to differentiation, survival, and tissue accumulation of mast cells in the tubulointerstitium (70, 71). Additionally, these effector T cells mediate recruitment of eosinophils and can activate B cells to produce IgE, which further enhances immune cell recruitment (72). Mast cells appear to be a critical source of TNF-α in allergic diseases, and urinary IL-9 and TNF-α are simultaneously elevated in human AIN (73–75). In the permissive environment of cytokines released from effector T cells and injured parenchymal cells, mast cells and eosinophils release proteases, leukotrienes, superoxides, and peroxidases to additionally perpetuate tissue injury (76, 77). Eosinophils also release major basic protein and eosinophilic cationic protein, which may have additional inflammatory actions (76). CXCL9, a chemokine released by many immune and nonimmune cells in response to IFN-γ, is an even more specific urinary marker for AIN than TNF-α and IL-9 (78). CXCL9 promotes lymphocyte recruitment at sites of inflammation through binding to its receptor CXCR3 and has a role in promoting kidney tubulointerstitial inflammation (78).

## Shared pathophysiology and response to injury

### Secondary immune-mediated injury.

Irrespective of the initial driver of tubular cell injury, there are preserved responses by the surviving tubular, endothelial, and interstitial cells that shape the trajectory of injury and subsequent repair responses. Immediately following injury, resident macrophages serve as a first line of defense against a robust immune response by cloaking sites of damage to prevent excessive immune cell recruitment and inflammatory cascades (79). In IRI mouse models, early injury of PTECs results in transcriptional upregulation of proinflammatory signaling pathways (e.g., ADAM17 and amphiregulin) which result in release of proinflammatory mediators (e.g., TNF-α, MCP-1, IL-6, IL-8, IL-34, and CXCL16) from injured TECs and ECs, overwhelming the resident mononuclear phagocytic cell-protective response (80–85). This leads to the infiltration of polymorphonuclear leukocytes (PMNs) and monocyte-derived proinflammatory macrophages, as well as lesser numbers of CXCR6^+^ T cells, to surround the injured tubules within the first 24 hours after injury (80, 86–90). B and T cells continue to infiltrate the kidney after AKI and contribute to ongoing inflammation in complex and subset-specific ways (91–95). CD4^+^ T cells are particularly important mediators of secondary immune-mediated injury, with Th1 and Th2 subsets exerting differential effects (93, 96, 97).

The tissue immune response is also important in animal models and humans with nephrotoxic tubular injury. For example, in the setting of bone marrow dyscrasias, activation of the STAT1 pathway during TEC metabolism of free light chains (FLCs) induces release of proinflammatory molecules IL-1β and TGF-β (98). Metabolism of monoclonal FLCs can also generate sufficient hydrogen peroxide to activate intracellular redox-sensitive signaling pathways, ultimately leading to cell death (99–102). In animal models of cisplatin-induced AKI, CXCL16 knockout inhibited infiltration of macrophages and T cells into the kidneys, reduced TEC apoptosis, and improved markers of kidney function (103). In oxalate nephropathy, oxalate crystals activate the NOD-like receptor family, pyrin domain containing 3 inflammasome (NALP3, NLRP3, or cryopyrin), resulting in release of IL-1β, macrophage infiltration, and progressive kidney failure (104). In an animal model of aristolochic acid nephropathy, macrophage-specific knockout of IRF4, a known regulator of macrophage migration and phenotype, led to reduced kidney macrophage infiltration following aristolochic acid administration and protection from injury (105).

In this early phase after injury, DAMP release by dying cells results in proinflammatory activation of recruited and resident immune cells, leading to further injury through release of ROS and caspase activation, which facilitates death of sublethally injured TECs. Prevention of this initial PMN and monocyte-derived macrophage infiltration and proinflammatory activation decreases the degree of injury and kidney function decline in animal models (90, 106–111). Proinflammatory M1 macrophages, induced by exposure to IFN-y, LPS, TNF-α, and IL-6 and expressing high levels of inducible nitric oxide synthase 2, IL-12, IL-23, and Ly-6C, appear to be the dominant macrophage phenotype in this initial injury-promoting phase (90).

### Mitochondrial dysfunction.

The loss of healthy mitochondria in PTECs likely accelerates ATP depletion, cellular injury, and fibrosis development after AKI (112, 113). The dependence on mitochondrial oxidative phosphorylation for ATP generation makes PTECs highly oxygen dependent. Insufficient oxygen delivery in the setting of AKI can cause mitochondrial dysfunction, ROS overproduction, and inflammation (114) and may also contribute to cell-cycle arrest (115). Furthermore, release of mitochondrial DNA by injured cells can induce TLR-dependent immune responses and kidney injury progression (116, 117).

### Cell-cycle arrest/senescence.

After injury, PTECs with DNA damage often undergo cell-cycle arrest at G2/M, which may be protective against genomic instability (118, 119). In several mouse models of acute injury, DNA damage activates a cell-autonomous DNA damage response that is normally protective against kidney function impairment and fibrosis (120). However, prolongation of cell-cycle arrest can lead to cellular senescence, considered an accelerated aging phenotype, that has been linked to a profibrotic secretory phenotype leading to kidney fibrosis (121–123). The earliest onset of cellular senescence occurs within 2 to 3 days after AKI and is reported to be mediated by epithelial TLRs and IL-1 receptors on TECs, with autocrine and paracrine spreading of senescent cells to the surrounding epithelium (124, 125). Therapeutic targeting of senescent cells after AKI has resulted in conflicting results to date (126, 127).

### Cell death.

Depending on the severity of initial injury and the degree of recruitment and activation of the innate immune response, multiple types of tubular cell death can occur. Apoptosis is a coordinated deconstruction and clearance of cellular components that typically minimizes plasma membrane rupture and thus limits DAMP release and the secondary inflammatory response (24). In contrast, necrotic cell death, regulated or unregulated, results in plasma membrane disruption and extensive release of DAMPs (24). DAMPs serve as strong activators of the innate immune system and drive continued tissue inflammation and injury in a process known as necroinflammation (24). Classical DAMPs are detected by multiligand receptors, such as the receptor for advanced glycosylation end products, NOD-like receptors, RIG-I-like receptors, and TLRs, and activate expression of multiple proteins, such as IL-6, TNF-α, and TGF-β, which serve as chemokines for continued recruitment of immune cells to the site of injury (41, 128). DAMP signaling activates the effectors of the innate immune system that are recruited to the site of injury, such as PMNs and macrophages, but simultaneously stimulates survival pathways in the injured TECs, such as autophagy (129). This aids in clearance of damaged intracellular organelles after injury to promote cell survival, but, when unchecked, may further stimulate ROS generation and lead to cell death instead.

The types of PCD that constitute necroinflammation have received increased attention in recent years in the setting of AKI. Necroptosis, mediated by the RIPK3/MLKL necrosome complex, results in permeabilization of the cell membrane and cell death. RIPK3 and MLKL are upregulated in PTECs after IRI, leading to increased NLRP3 inflammasome activation and IL-1β secretion from proinflammatory macrophages, which in turn increases necrosome formation in PTECs to generate an uncontrolled inflammatory loop (130). Inhibiting necroptosis improved renal outcomes in several animal AKI models (131, 132). Mitochondrial damage also resulted in release of ROS, mitochondrial DNA (mtDNA), and cardiolipin, which activated the NLRP3 inflammasome, upregulated IL-1β and IL-18, and sustained chronic inflammasome activation after AKI (133).

Other modes of PCD have also been implicated in AKI. Ferroptosis, driven by oxidative stress and iron-dependent phospholipid peroxidation, manifests as a loss of cell membrane integrity and blebbing, shrinking mitochondrial cristae, and increased mitochondrial bilayer density. Ferroptosis may mediate TEC death in rhabdomyolysis-associated AKI, where accumulated myoglobin in the kidney is metabolized to release large amounts of iron (134, 135). Ferroptosis may also occur after various nephrotoxin exposures and after IRI, particularly in the reperfusion phase, which includes excessive ROS production, cascade-amplified inflammatory reactions, and ferritinophagy (136–139) (Figure 2).

Pyroptosis, activated by inflammation-related caspases that cleave gasdermin, can be induced in some types of AKI. Cleaved gasdermin translocates to the membrane and creates pores that permit massive release of intracellular contents into the extracellular space. Increased expression of pyroptosis-triggering caspases was found after IRI and cisplatin-induced AKI, but this pathway may be most consequential in sepsis-induced AKI (140–143) (Figure 2). In animal models, LPS administration can activate the caspase-1/IL-1β pathway during AKI to initiate pyroptosis, and inhibition of pyroptosis is protective in animal models of septic shock (142, 143).

## Kidney recovery

**Figure 3:**
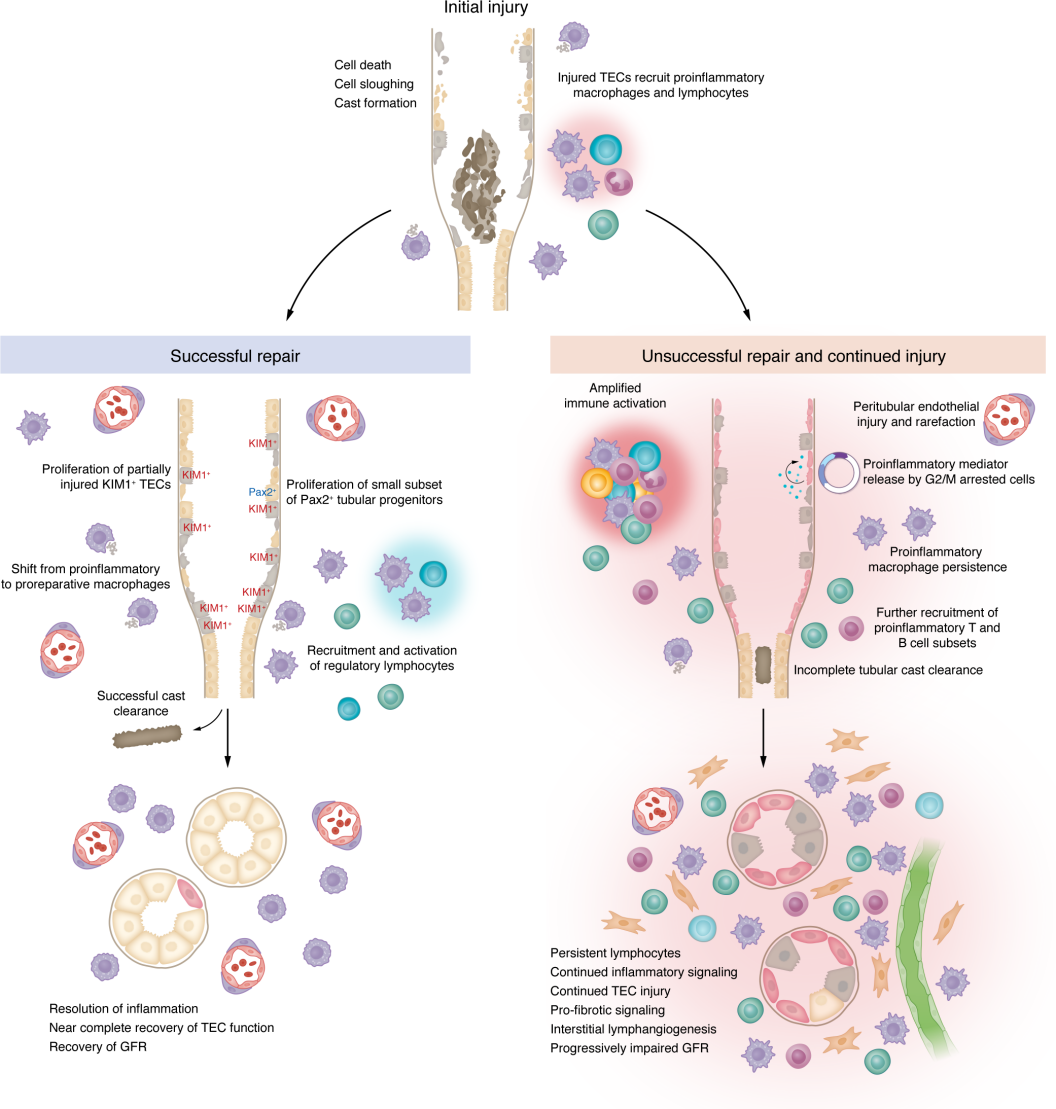
Resolution of AKI. Immediately following injury, dying cells form casts in the tubular lumen in association with Tamm-Horsfall protein while surviving TECs recruit proinflammatory macrophages and lymphocytes to the tubulointerstitium. (Left) With successful repair and recovery after AKI, casts are cleared and proinflammatory macrophages shift to a proreparative phenotype that promotes TEC proliferation and dampens the immune response, allowing TECs to redifferentiate and restore tubule architecture and function. A small subset of Pax2^+^ tubular progenitors also contribute to regeneration of necrotic epithelial regions (199). (Right) If injury to a particular tubule or region is severe or sustained, the local immune response is amplified with enhanced recruitment of proinflammatory T cells and B cells into the interstitium and persistence of proinflammatory macrophage populations. This can lead to tubular cell G2/M arrest and adoption of a senescence-associated secretory phenotype with release of inflammatory cytokines, growth factors, proteases, and immune modulators that recruit additional proinflammatory macrophages and lymphocytes that sustain the local inflammatory response and can lead to secondary injury to adjacent tubules. This persistent inflammatory milieu promotes prolymphangiogenic signaling, ongoing TEC injury, profibrotic signaling, and progressive impairment of GFR.

While secondary proinflammatory immune responses contribute to the overall degree of cellular injury in AKI, the subsequent coordinated spatiotemporal transition of this immune response is also critical to successful tubular repair (Figure 3). After the wave of proinflammatory M1 macrophage and PMN infiltration into the kidney in the first 24 hours after injury, PMNs egress and macrophages transition to an alternatively activated, proreparative phenotype beginning by day 2 (80, 87, 89, 90, 144). In murine models of IRI, expression of arginase-1 by transitioning macrophages in response to CSF-2 expressed by tubular cells was required for maximal TEC proliferation, which underlies kidney repair after injury (144, 145). CSF-1, the principal macrophage growth and survival factor, is released by surviving TECs to enable macrophage survival during the transition to a reparative phenotype and also promotes proreparative effects through direct autocrine and paracrine action on TECs (87, 146, 147). Macrophage-derived IL-22, retinoic acid receptor ligands, and Wnt/β-catenin signaling also play a role in stimulating repair and regeneration of TECs after ischemic injury (148–151). With successful repair after injury, infiltrated macrophages either egress or undergo apoptosis, whereas more severe or unresolved injury results in macrophage persistence adjacent to injured tubules with transition to a profibrotic phenotype that promotes the activation of interstitial myofibroblasts (152–154). Resident macrophages are additionally critical in the late phases of repair, where they act as scavengers of apoptotic cells and regulate kidney-infiltrating T cells (155–157).

Beyond the well-known proinflammatory and tissue-damaging roles of lymphocytes in AKI, subsets of T cells are recognized to play an antiinflammatory role assisting in recovery after AKI. Foxp3^+^CD4^+^ Tregs and TCR^+^CD4^–^CD8^–^ double-negative (DN) T cells are two such subsets, with expansion of Treg or DN T cell populations promoting kidney recovery after AKI and Treg depletion aggravating dysfunction (158–161). Similarly, while B cells and plasma cells contribute to inflammation after AKI through production of immunoglobulins and subsequent engagement of cellular immunity and potential recruitment of the complement system, a subset of regulatory B cells produce IL-10 and are antiinflammatory in other disease models, and may be protective after AKI (91, 162, 163). Finally, the ECs of the repairing kidney may substantially contribute to kidney recovery after AKI through regulating inflammatory responses or by providing proreparative signals (164–167).

After ATI, brisk replication by surviving TECs replaces lost TECs and restores tubular architecture (168, 169). The replacement of lost TECs involves both cell-autonomous survival responses and reversal of the proinflammatory innate immune response. During the initial injury phase, surviving TECs shed their brush border and downregulate transporter expression. These dedifferentiated cells, often KIM-1^+^ and *Sox9*^+^, acquire a proliferative phenotype and constitute the bulk of cells that undergo division to replace the lost TECs (169–171). In proliferating PTECs, the transcription factor *Foxm1* was induced early in injury following epithelial growth factor receptor (EGFR) stimulation and was required for epithelial proliferation in vitro, suggesting that EGFR/FOXM1-dependent signaling is required for PTEC proliferative repair (172). *Sox9* deactivation after epithelial repair was also required to prevent WNT pathway–induced fibroproliferative effects and chronic injury (173). It is well established that tubular epithelium has a limited regenerative potential after cellular loss, which may impact the degree of repair that is possible in cases of severe injury or in older individuals (174, 175).

KIM-1 expression has been used as an injury marker in AKI, as it is highly upregulated on the surface of injured kidney TECs. KIM-1–expressing PTECs in animal models and cell-line studies acquire attributes of semiprofessional phagocytes, with the specific ability to recognize apoptotic cell-surface–specific epitopes (176). KIM-1 is also suggested to facilitate an important immunomodulatory role of PTECs through involvement in antigen presentation facilitated by MHC I and II, leading to the suppression of CD4^+^ T cell activation and an increase in Treg recruitment (177). Thus, upregulation of KIM-1 is likely to be an important component of the injured PTEC survival response.

## AKI to CKD transition

Even a single AKI episode is associated with an increased risk of CKD (178, 179). In humans and animal models, an episode of AKI due to TEC injury often leads to unresolved injury of some tubules, even when markers of GFR return to baseline values, a risk that increases with age (180, 181). While exact mechanisms underlying the AKI-to-CKD transition remain incompletely understood, crosstalk between these chronically injured, or failed-repair, tubules and the immune system appears to play a prominent role (84, 182). The largest molecular reference atlas of the human kidney to date (including >400,000 cells or nuclei from 35 reference tissue donors and 36 AKI and CKD tissue donors) demonstrated several tissue myeloid and lymphoid immune cell populations in accordance with previous kidney atlas studies (183–186). Mutually exclusive niches enriched in either myeloid or T cells were identified, with myeloid-rich neighborhoods associated with “adaptive” TEC states and T cell–rich neighborhoods associated with “degenerative” TEC states (183). In patients with AKI, T cell and neutrophil numbers negatively correlate with recovery of estimated GFR (84). Animal studies suggest that macrophages persisting beyond the initial repair phase may promote the development of a neutrophil and T cell–mediated proinflammatory environment contributing to progressive tubule damage. In a study comparing mice subjected to unilateral IRI with either contralateral nephrectomy (where tubule repair predominates) or contralateral kidney intact (where tubule atrophy predominates), kidneys undergoing atrophy had more macrophages with higher expression of homing chemokines, correlating with a second wave of proinflammatory neutrophil and T cell recruitment and increased tubular injury (84). When PMNs and T cells were depleted after day 5, the late tubule atrophy response was reduced.

While successful kidney recovery after AKI is marked by rapid proliferation of many sublethally injured TECs, some injured TECs instead undergo cell-cycle arrest in the G2 phase and maintain persistently lower expression of solute and solvent transporters (187). In these TECs, specific transcriptional programs (*Snai1*, *Twist1*) promote TGF-β1 induction of cell-cycle arrest (187). G2/M cell-cycle arrest both limits cellular repair and regenerative potential and leads to the development of a pathologic secretome that promotes a proinflammatory and profibrotic environment (121, 187). This is thought to be particularly relevant in severe as compared to moderate ATI, and its physiologic purpose is incompletely understood (121).

VCAM-1–expressing PTECs, identified as “failed repair” or “late injured” PTECs, also express a proinflammatory and profibrotic phenotype predictive of tubular atrophy (83, 84, 188, 189). As a marker of atrophic and CKD transitioning tubules, VCAM-1 demonstrates a later onset of expression compared with other common tubule injury markers such as KIM-1 (21, 84, 188). VCAM-1 expression is induced through NF-κB–dependent TNF-α and IL-1β signaling and mediates increased immune cell adhesion to TECs to promote further tubular injury (190). This may underlie the formation of tertiary lymphoid tissues, ectopic lymphoid structures that portend a poor renal outcome and are prominent near injured PTECs in models of AKI in aging (83, 191).

Growth of lymphatic vessels within the kidney, or lymphangiogenesis, has been associated with numerous forms of kidney disease, including AKI, and is strongly associated with interstitial fibrosis in CKD (192, 193). The strongest prolymphangiogenic signaling molecules, VEGF-C and -D, are secreted by renal TECs and macrophages after AKI (194). Recent single-cell RNA-Seq (scRNA-Seq) data in IRI and cisplatin-induced injury mouse models suggest that lymphatic ECs may actively shape the immune response to AKI (195). Renal lymphatic ECs demonstrated changes in expression of lymphangiogenic signaling pathways, major histocompatibility complex genes, and genes involved in T cell differentiation, antigen presentation, and cytokine signaling (195). The role of the lymphatics and lymphangiogenesis in promoting and/or sustaining tubulointerstitial disease remains incompletely explored with conflicting published data, highlighting the need to increase the research focus on this aspect of the immune response in kidney injury and repair.

## Future directions

Despite a vast body of research, the management of TEC injury in AKI has remained virtually unchanged for decades, and options for disease-modifying therapies are nonexistent. One challenge to the successful targeting of AKI therapies is in the timing of delivery. Numerous therapies delivered prior to AKI have shown promise in animal settings; however, their translation to clinical practice has proven difficult. Using a functional biomarker such as creatinine to define AKI imposes a delay in the diagnosis of AKI and makes the clinical application of a treatment that is intended to be delivered concurrent with AKI clinically impractical. A second factor underlying the lack of successful translation of findings from preclinical studies to clinical therapies is the use of simplified laboratory models of AKI in healthy young animals under controlled conditions, when clinical AKI typically occurs in older patients with complex medical histories and exposures. Furthermore, while they are an irreplaceable tool in scientific discovery, the best-matched animal models have an immune repertoire and responses that differ from humans in potentially important ways (196–198).

The recent advent of both dissociated and spatially resolved single cellular modalities allowing for the robust, specific interrogation of proteomic, transcriptomic, epigenomic, and metabolomic features of human kidney biopsies holds enormous potential for discovery. Integration of the vast amount of such data, which is quickly becoming accessible, will lead to improved insights into the molecular patterns being activated in human TEC injury states and thus allow us to move away from simplistic classifications of AKI as “pre-renal” and “ATI” and instead group kidney injury responses mechanistically based on cellular responses and trajectories. Such data integration is a goal of the Kidney Precision Medicine Project (KPMP), an ambitious, multi-year project funded by the National Institute of Diabetes and Digestive and Kidney Diseases (NIDDK), which pools expertise and resources across many institutions to harness this new frontier of discovery toward advancing our knowledge and treatment options for AKI.

## Version 1 03/17/2025

Electronic publication
